# Assessing the Correlation of Rate of Pathological Complete Response and Outcome in Post Neoadjuvant Chemotherapy Setting and Molecular Subtypes of Breast Cancer

**DOI:** 10.7759/cureus.37449

**Published:** 2023-04-11

**Authors:** Ahmad Omair, Abdulmohsen Alkushi, Ghaida Alamri, Talal Almojel, Sara Alsadun, Emad Masuadi, Haitham Arabi, Amin E Mohamed, Omalkhair A Abulkhair

**Affiliations:** 1 Pathology, College of Science and Health Professions, King Saud bin Abdulaziz University for Health Sciences, Riyadh, SAU; 2 Pathology, King Abdullah International Medical Research Center (KAIMRC), Riyadh, SAU; 3 Pathology, King Abdulaziz Medical City, Riyadh, SAU; 4 Pathology, College of Medicine, King Saud bin Abdulaziz University for Health Sciences, Riyadh, SAU; 5 Medicine, College of Medicine, King Saud bin Abdulaziz University for Health Sciences, Riyadh, SAU; 6 Medicine, King Abdullah International Medical Research Center (KAIMRC), Riyadh, SAU; 7 Pathology, King Saud University, Riyadh, SAU; 8 Surgery, King Abdulaziz Medical City, Riyadh, SAU; 9 Research Unit/Biostatistics, College of Medicine, King Saud bin Abdulaziz University for Health Sciences, Riyadh, SAU; 10 Research Unit/Biostatistics, King Abdullah International Medical Research Center (KAIMRC), Riyadh, SAU; 11 Oncology, King Abdulaziz Medical City, Riyadh, SAU; 12 Oncology, Dr. Sulaiman Al Habib Hospital, Riyadh, SAU

**Keywords:** overall survival, disease-free survival, pathological complete response, neoadjuvant chemotherapy, breast cancer

## Abstract

Background

Neoadjuvant chemotherapy (NAC) is being widely used in treating breast cancer (BC). This study aimed to analyze the correlation between clinicopathological features, immunohistochemistry (IHC)-based molecular subtypes, and the pathological response to NAC and its relationship with disease-free survival (DFS) and overall survival (OS).

Materials and methods

A retrospective analysis of 211 breast cancer patients who received NAC between 2008 and 2018 was performed. Tumors were classified by IHC into luminal A, luminal B, human epidermal growth factor receptor 2 (HER2)-enriched, and triple-negative subtypes. The chi-square test was used to assess the association between pathological response and clinicopathological parameters. Cox regression analysis was used to assess factors related to DFS and OS.

Results

Post NAC, 19.4% of patients achieved a pathologic complete response (pCR). Estrogen receptor (ER), progesterone receptor (PR), HER2 (p<0.001, 0.005, and 0.02), Ki67 (p=0.03), molecular subtypes (p<0.001), T stage (p=0.04), and N stage (p=0.01) were significantly associated with pathological response. The rate of pCR was highest among HER2-enriched and triple-negative tumors (45.2% and 28%, respectively) with OR=0.13 and p<0.001 for the HER2-enriched subtype. Patients with pCR were 61% less likely to develop metastasis (adjusted hazard ratio [aHR]=0.39, p=0.06, 95% CI=0.14-1.06) and were significantly associated with better OS (aHR=0.07, p=0.02, 95% CI=0.01-0.61). Patients who were ≤40 years old (aHR=2.1, p=0.01), with T4 (aHR=3.4, p=0.02), grade 3 (aHR=2.5, p=0.01), and node-positive disease (HR=2.24, p=0.02) were at an increased risk of developing metastasis. High Ki67 was found to be significantly associated with better DFS (p=0.006).

Conclusion

HER2-enriched and triple-negative BC were associated with a higher rate of pCR. Patients with pCR had significantly better DFS and OS. Younger age, advanced stage, higher grade, and lymph node involvement were risk factors for metastasis.

## Introduction

Breast cancer (BC) is the most common type of cancer affecting women worldwide, and it is the second leading cause of female cancer-related deaths [1]. The management of BC varies depending on the stage of the disease and its biological behavior and involves a multidisciplinary team approach [2]. Systemic therapy has mainly been used after surgical treatment in BC patients; however, recent studies have shown that adjuvant and neoadjuvant chemotherapies (NAC) are equally effective [1, 2]. The type of NAC is mainly tailored according to the pathological sub-types, tumor biology, and extent of disease at presentation [1]. It has traditionally been the standard therapy in patients with locally advanced BC (LABC) and inflammatory BC, allowing for a reduction in tumor size and, therefore, optimizing surgical resection. From a clinicopathological perspective, LABC represents a relatively variable group of tumors. In recent years, the use of molecular classification (MC) of BC has significantly improved the understanding of breast carcinogenesis and offered an opportunity for improving therapeutic intervention. As the application of gene expression profiling (GEP) in daily practice is neither economical nor practical, many studies have proposed immunohistochemistry (IHC)-based MC to study BC, which has shown a comparable predictive and prognostic value [3]. Using estrogen receptor (ER), progesterone receptor (PR), human epidermal growth factor receptor 2 (HER2), and Ki-67 immunostains, BC can be generally categorized into four major IHC-based molecular subtypes, including luminal (A and B), HER2-enriched, and triple-negative subtypes. Luminal A tumors often have an IHC profile of high ER and PR expression, negative HER2, and low Ki-67. On the other hand, luminal B (luminal B HER2-negative) tumors have a positive expression of ER and PR and a negative expression of HER2 with higher Ki67. HER2-positive tumors are divided into two subtypes: luminal B HER2-positive subtype with positive expression of ER, PR, and HER2 (triple positive BC) and HER2-enriched subtype with negative expression of ER and/or PR and positive expression of HER2. The last subtype is triple-negative BC which is defined by the lack of expression for ER, PR, and HER2 by IHC analysis [3]. These IHC-based molecular subtypes vary based on each subtype’s biological properties, including their spectrum of clinical features, response to the treatment, and prognosis.

NAC has been used to improve tumor resection by downstaging the primary tumor and identifying patients who display a pathologic complete response (pCR) [4, 5]. pCR is used as a parameter to assess the prognosis of malignancy, invasiveness of cancer, and evaluate the effectiveness of new therapeutic agents in neoadjuvant clinical trials. There are multiple definitions of pCR given in the literature [5]. However, they mainly focus on the level of invasiveness of carcinoma in the breast with or without the involvement of lymph nodes (LN) and the presence or absence of ductal carcinoma in situ (DCIS), upon completion of NAC, depending on the used system of evaluation [4, 5]. Most systems consider the absence of residual LN disease as pCR since its presence usually correlates with a worse prognosis [5]. One of the definitions of pCR is the disappearance of both invasive carcinoma and DCIS with the absence of LN invasion, e.g., the Chevalier system and Pinder classification [5, 6]. Another definition is the absence of invasive carcinoma and lymph node invasion regardless of the presence of DCIS, e.g., American Joint Committee on Cancer (AJCC) [5]. Others defined it as the absence of invasive carcinoma regardless of the presence or absence of DCIS or LN invasion, e.g., Miller-Payne Grading System [5]. A less commonly used definition is the absence of invasive carcinoma with or without DCIS and a few scattered residual tumor cells. The most commonly used is the absence of invasive tumors in the breast and axilla with or without the presence of DCIS. Pathological partial response (pPR) is defined as the presence of residual invasive carcinoma with stromal alterations. Pathological no response (pNR) is identified when there is little modification in the original tumor appearance [6]. Achievement of a pCR in the breast and LN after NAC has been confirmed to increase disease-free survival (DFS) and improve the patient’s outcome indicating that it is an excellent prognostic indicator [7, 8]. In comparison to hormone receptor (HR)-positive subtypes, the HR-negative subtypes (HER2-enriched and triple-negative) have increased chemosensitivity. They are more likely to achieve pCR, increasing the indication of NAC in these subtypes [9]. Higher rates of pCR can be achieved when selecting the appropriate and tailored management regimens for each specific type of BC [10]. This study aimed to identify and analyze the correlation of the clinicopathological features and IHC-based molecular subtypes of BC tumors with the pathological response to NAC and its relationship with patients’ outcomes.

## Materials and methods

Study population

This study is a single-center retrospective study conducted at King Abdulaziz Medical City (KAMC), a tertiary care center in Riyadh, Kingdom of Saudi Arabia. The study was approved by the Institutional Review Board (IRB), with the approval number RC19/237/R. It included a retrospective chart review of all BC patients who received NAC between 2008 and 2018. Through a non-probability consecutive sampling approach, a total of 211 female patients who were diagnosed with BC and received NAC were included. Patients who had excisional biopsies prior to receiving the NAC were excluded.

Data collection

Patients' information was collected manually by the co-investigators, then inserted into a Microsoft Excel sheet. The data included the patient's demographic information, date of diagnosis, tumor cell type, pathological parameters (tumor grade, Ki67, ER, PR, and HER2 status), TNM stage, pathological response status (complete [pCR], partial [pPR], or no response [pNR]), date of the last contact, vital status at last follow-up (dead, alive), date and cause of death, recurrence status (disease-free, local, metastatic), date of recurrence, and location of metastasis. HER2 status is assessed initially by IHC adopting American Society of Clinical Oncology/College of American Pathologists (ASCO/CAP) guidelines followed by reflex Fluorescent in Situ Hybridization (FISH) testing for the 2+ results. These variables were obtained by the study group from the hospital's digital medical records, as well as the oncology and pathology reports were then reviewed and entered into the data collection sheet. 

Pathological response definition

In this study, the pathological response was defined as pCR when there was an absence of invasive tumor in the breast and axilla with or without the presence of DCIS; pNR, when little modification was noted in the original tumor by the pathological assessment on a surgical specimen, and the presence of residual invasive carcinoma in either breast or axilla was defined as pPR.

IHC-based molecular classification

According to the IHC-based molecular classification [3] and based on four immunohistochemical markers (ER, PR, HER2, and Ki67), the tumors were classified into luminal A, luminal B, HER2-enriched, and triple-negative subtypes. Luminal A tumors had positive ER and PR expression and negative expression of HER2 with lower Ki67 (≤14%). Luminal B tumors had either a positive expression of ER and PR, negative expression of HER2 with higher Ki67 (>14%), or positive expression of ER, PR, and HER2 (triple positive breast cancer) regardless of the Ki67 level. HER2-enriched subtype had a negative expression of ER and/or PR and positive expression of HER2. Triple-negative tumors showed negative expression of the three markers regardless of the Ki67 level. 

Statistical analysis

Data were entered into Microsoft Excel and then analyzed using the statistical program IBM SPSS Statistics for Windows, Version 24.0 (IBM Corp., Armonk, NY). Categorical data were presented as frequencies and percentages and as mean ± SD for numerical data. The chi-square and Fisher's exact tests assessed the association between treatment response and clinicopathological parameters. Ordinal logistic regression was used to obtain the adjusted odds ratio and treatment response. Cox regression analysis was performed to assess factors related to DFS and factors related to overall survival (OS). The level of statistical significance was set at ≤ 0.05.

## Results

The clinicopathological characteristics of the study population are summarized in Table 1.

**Table 1:** Clinicopathological characteristics of the study population. ER: Estrogen receptor; PR: Progesterone receptor; HER2: Human epidermal growth factor receptor 2.

Baseline characteristics	Count (N)	N%
Age (years)		
≤ 40	45	21.3%
> 40	166	78.7%
Tumor Grade		
1 and 2	98	46.4%
3	113	53.6%
ER Expression		
Positive	128	60.7%
Negative	83	39.3%
PR Expression		
Positive	100	47.4%
Negative	111	52.6%
HER2 Status		
Positive	71	33.6%
Negative	140	66.4%
IHC-based molecular subtype		
Luminal A	41	19.4%
Luminal B	89	42.2%
HER2-enriched	31	14.7%
Triple-negative	50	23.7%
Tumor T stage		
1	18	8.6%
2	124	59.0%
3	37	17.6%
4	31	14.8%
Tumor N stage		
0	79	37.4%
1 or more	132	62.6%
Pathological Response		
Complete	41	19.4%
Partial	142	67.3%
No response	28	13.3%

The mean age ± SD of the patients was 48.6 ± 11.0 years (range: 25-82). Most patients (78.7%) were >40 years old compared to 21.3% who were ≤40. Most patients (53.6%) had grade 3 tumors, whereas out of the remaining 46.4% cases, only 13 patients (6.2%) had grade 1 tumors. Luminal B was the most common IHC-based molecular subtype identified (n=89; 42.2%), followed by triple-negative (n=50; 23.7%) and luminal A (n=41; 19.4%), and HER2-enriched (n=31; 14.7%) was found to be the least common subtype. Tumor stage 2 (T2) and nodal stage 1 (N1) were the most frequently recognized TNM stages (59% and 52.6%, respectively). Moreover, most of the tumors were ER-positive (60.7%), PR-negative (52.6%), and HER2-negative (66.4%). The mean Ki67 was 40.9 ± 26.7 with a range of 5-95. Most patients achieved pPR (67.3%) post-NAC, followed by pCR (19.4%) and pNR (13.3%). The mean follow-up duration of our patients was 5.8 ± 3.0 years (range: 0.7-17.4). Univariate analysis for correlation between pathological response and clinicopathological parameters (Table 2) revealed that ER expression (p <0.001), PR expression (p=0.005), HER-2 status (p=0.02), IHC-based molecular subtypes (p <0.001), tumor T stage (p=0.04), tumor N stage (p=0.01), and Ki67 (p=0.03) were significantly associated with tumor pathological response. Tumor grade showed a tendency towards significance (p=0.056), and age was the only parameter that was found to be non-significant (p=0.80). Among the significant factors, a higher percentage of cases with ER and PR negative, HER2 positive, triple negative, and HER2-enriched subtype, tumor stage 2, no LN involvement, and greater Ki67 were found to achieve a pCR. Among luminal A tumors, three (7.3%) achieved pCR, 29 (70.7%) achieved pPR, and nine (22%) had pNR. Among luminal B tumors, 66 (74.2%) achieved pPR, and only 13 (14.6%) and 10 (11.2%) had pNR and pCR, respectively. Among HER2-enriched tumors, 14 (45.2%) achieved pCR, 17 (54.8%) achieved pPR, and none had pNR. Among the triple-negative tumors, 14 (28%) achieved pCR, 30 (60%) achieved pPR, and six (12%) had pNR (Table 2).

**Table 2:** Univariate analysis for correlation between clinicopathological parameters and pathological response. ER: Estrogen receptor; PR: Progesterone receptor; HER2: Human epidermal growth factor receptor2. ^ Fisher's exact test.

Variables	Complete Response	Partial Response	No Response	P-value
	n (%)	n (%)	n (%)	
Age (years)				0.81
≤ 40	10 (22.2)	30 (66.7)	5 (11.1)	
> 40	31 (18.7)	112 (67.5)	23 (13.9)	
Tumor Grade				0.056^
1 and 2	13 (13.3)	68 (69.4)	17 (17.3)	
3	28 (24.8)	74 (65.5)	11 (9.7)	
ER Expression				<0.001
Positive	13 (10.2)	94 (73.4)	21 (16.4)	
Negative	28 (33.7)	48 (57.8)	7 (8.4)	
PR Expression				0.005
Positive	10 (10.0)	75 (75.0)	15 (15.0)	
Negative	31 (27.9)	67 (60.4)	13 (11.7)	
HER2 Status				0.02
Positive	21 (29.6)	44 (62.0)	6 (8.5)	
Negative	20 (14.3)	98 (70.0)	22 (15.7)	
IHC-based molecular subtype				<0.001
Luminal A	3 (7.3)	29 (70.7)	9 (22.0)	
Luminal B	10 (11.2)	66 (74.2)	13 (14.6)	
HER2-enriched	14 (45.2)	17 (54.8)	0 (0.0)	
Triple-negative	14 (28.0)	30 (60.0)	6 (12.0)	
Tumor T stage				0.04^
1	2 (11.1)	16 (88.9)	0 (0.0)	
2	27 (21.8)	85 (68.5)	12 (9.7)	
3	6 (16.2)	24 (64.9)	7 (18.9)	
4	6 (19.4)	16 (51.6)	9 (29.0)	
Tumor N stage				0.01
0	22 (27.8)	52 (65.8)	5 (6.3)	
1 or more	19 (14.4)	90 (68.2)	23 (17.4)	
Ki67 mean (SD)	48.9 (26.3)	40.5 (26.8)	31.6 (24.2)	0.03

Table 3 shows the ordinal logistic regression analysis to analyze the factors associated with pathological response.

**Table 3:** Ordinal logistic regression of factors associated with pathological response. * Reference group; HER2: Human epidermal growth factor receptor 2; Dependent variable: Response: Complete, Partial, No Response (reference group).

Parameter	P-value	OR	95% CI for OR	
			Lower	Upper
Age (years)				
≤ 40	0.82	0.92	0.45	1.90
> 40*		1		
Tumor Grade				
1 and 2	0.55	1.28	0.56	2.81
3*		1		
IHC-based molecular subtype				
Triple-negative	0.16	0.40	0.11	1.44
HER2-enriched	<0.001	0.13	0.04	0.43
Luminal B	0.64	0.79	0.31	2.06
Luminal A*		1		
Ki67	0.76	0.99	0.98	1.01

The dependent variable in this model was the pathological response, where pNR was used as a reference group. Similarly, among other factors, age > 40 years, grade 3, and luminal A subtype were used as a reference group for comparison. Compared to the rest of the molecular subtypes, only the HER2-enriched subtype displayed significant association (p=<0.001) and was less likely to have an outcome of pNR compared to luminal A (OR=0.13, 95% CI=0.04-0.43). Prior to NAC, none of the patients in the study population had metastasis; however, 33.2% developed metastasis during the follow-up period (range: 0.7-17.4 years). The sites of metastases were lung, LNs (other than axillary), bone (n=37; 17% for each, respectively), liver (n=34; 16%), brain (n=15; 7%), and other sites (n=55; 43%). Some patients developed metastasis in more than one site. Table 4 demonstrates the factors related to DFS.

**Table 4:** Multivariable Cox regression analysis of factors related to disease-free survival. aHR: Adjusted hazard ratio; HER2: Human epidermal growth factor receptor 2; * Reference group

Parameter	P-value	aHR	95% CI for aHR	
			Lower	Upper
Age (years)				
≤ 40	0.01	2.10	1.2	3.69
> 40*		1		
Grade				
3	0.01	2.50	1.23	5.05
1 and 2*		1		
Ki67	0.006	0.98	0.97	0.99
IHC-based molecular subtype				
Triple-negative	0.10	2.49	0.83	7.47
HER2-enriched	0.32	1.75	0.58	5.28
Luminal B	0.23	1.65	0.73	3.72
Luminal A*		1		
Tumor T stage				
4	0.02	3.40	1.17	9.85
3	0.90	1.07	0.36	3.20
2	0.72	1.20	0.45	3.14
1*		1		
Tumor N stage				
1 or more	0.02	2.24	1.16	4.31
0*		1		
Pathological Response				
Complete	0.06	0.39	0.14	1.06
Partial	0.92	0.97	0.49	1.91
No response*		1		

**Figure 1:**
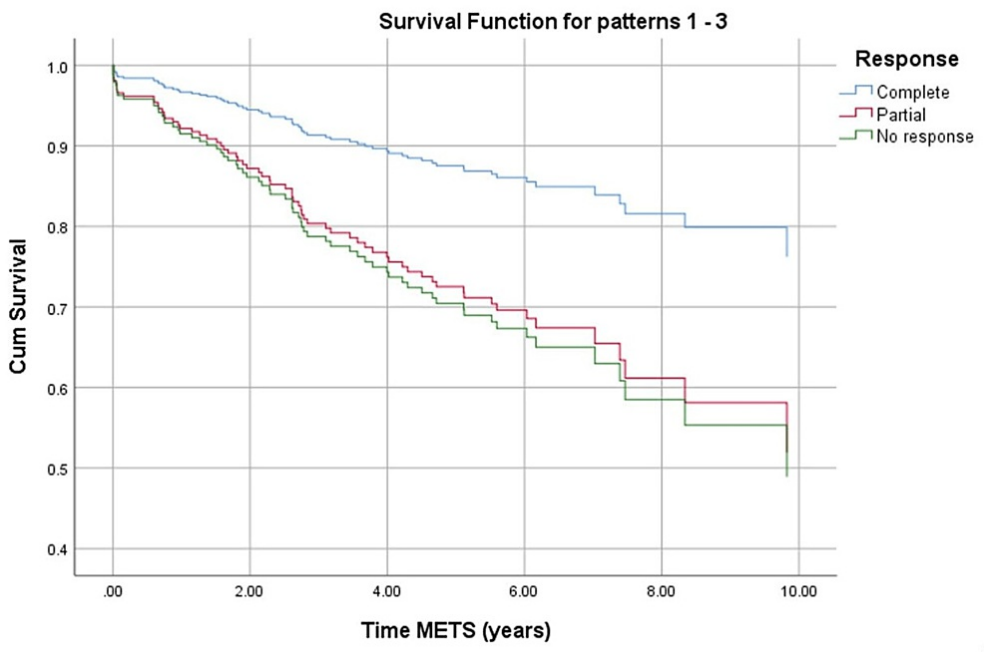
Survival curve for metastasis (METS) stratified by the pathological response (Cox regression analysis).

Patients who achieved a pCR (aHR=0.39) were 61% less likely to develop metastasis (p=0.06) than those with pNR. Ki67 was significantly associated with DFS (p=0.006). With every 10% increase in Ki67, the chance of developing metastasis decreases by 20% (aHR=0.98). Compared to the reference group, patients with ≤40 years of age (aHR=2.10; p=0.01), tumor stage 4 (T4) (aHR=3.40; p=0.02), grade 3 (aHR=2.50; p=0.021), and LN-positive disease (aHR=2.24; p=0.02) were at a significantly increased risk of developing metastasis. None of the molecular subtypes was found to be a significant risk factor for developing metastasis. Based on the Cox regression analysis in Table 4, Figure 1 presents the DFS curves stratified by the pathological response and shows that patients with pCR had better DFS (longer time to metastasis) than pNR (p=0.05).

Table 5 illustrates the OS of patients.

**Table 5:** Multivariable Cox regression analysis of factors related to overall survival. aHR: Adjusted hazard ratio; HER2: Human epidermal growth factor receptor 2; * Reference group

Parameter	P-value	aHR	95% CI for aHR	
			Lower	Upper
Age (years)				
≤ 40	0.15	1.80	0.81	4.00
> 40*		1		
Grade				
3	0.12	2.42	0.80	7.35
1 or 2*		1		
Ki67	0.08	0.98	0.96	1.00
IHC-based molecular subtype				
Triple-negative	0.11	3.20	0.77	15.19
HER2-enriched	0.42	2.00	0.37	10.78
Luminal B	0.74	1.22	0.38	3.95
Luminal A*		1		
Tumor T stage				
4	0.09	3.59	0.83	15.42
3	0.91	1.08	0.26	4.55
2	0.87	0.89	0.23	3.51
1*		1		
Tumor N stage				
1 or more	0.65	1.24	0.50	3.09
0*		1		
Pathological Response				
Complete	0.02	0.07	0.01	0.61
Partial	0.32	0.62	0.24	1.60
No response*		1		

**Figure 2:**
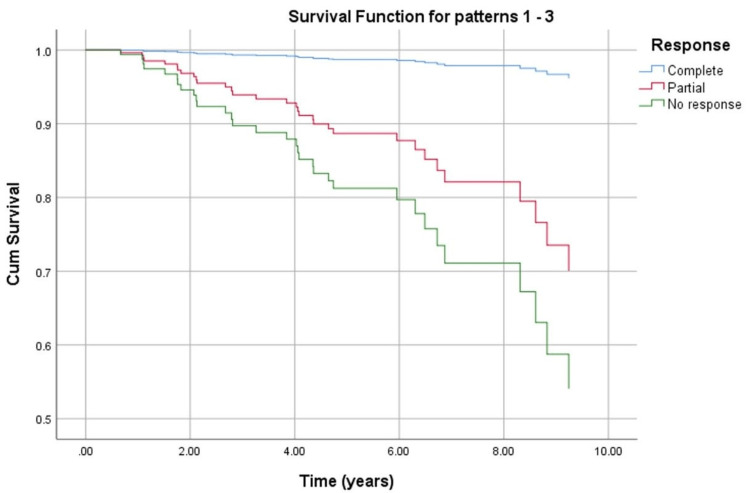
Patients' overall survival curve stratified by the pathological response (Cox regression analysis).

Patients with pCR (aHR=0.07) were at 94% less risk for mortality than those with pNR (p=0.02). Ki67 and tumor stage 4 showed a tendency towards association (0.08 and 0.09, respectively), where every 10% increase in Ki67 decreases the likelihood of mortality by 20% (aHR=0.98), and the chance of mortality increases by 3.6-fold with tumor stage 4 compared to stage 1 (aHR=3.59). Patients who were ≤40 years old had a higher risk of mortality (aHR=1.8), but it was not statistically significant (p=0.15). Based on molecular subtypes, no statistically significant association was observed for any subtype with mortality. However, cases with triple-negative tumors had a higher risk of mortality (aHR=3.20), followed by HER2-enriched (aHR=2.0) and luminal B (aHR=1.22) when compared to luminal A. Figure 2 illustrates OS curves based on Cox regression analysis (presented in Table 4), stratified by the pathological response, and shows that patients with pCR had better OS than pNR (p=0.01). Although non-significant (p=0.26), pPR was also observed to have better OS than pNR.

## Discussion

Although initially developed to treat large tumors that are neither operable at presentation nor require extended radical surgery [11], NAC is being increasingly utilized to down-stage breast tumors and potentially make a mastectomy-requiring patient eligible for breast-conserving surgery, leading to a better outcome in BC patients receiving NAC compared to those who did not [8]. pCR following NAC certainly highlights the tumor's sensitivity to therapy, but it is the predictability of long-term outcomes in terms of recurrence and survival that can validate its efficacy. Many clinical trials have shown that pCR relates to favorable clinical outcomes and could be used as a surrogate marker of better survival [10]. Therefore, this study focused on identifying clinicopathological parameters that affect the pCR and its association with outcome. Along with revealing the association of pCR with negative HR and positive HER2 status, early tumor stage, no nodal involvement, higher Ki67 index, and molecular subtypes (HER2-enriched and triple-negative), this study also showed pCR to be a positive predictor for OS and DFS, thus strengthening the efficacy of NAC in LABC. The mean age of our study population was 48.6 years (SD±11.0), which is relatively younger than the reported cases worldwide but similar to what has been reported by the Saudi Arabian Cancer Incidence Report and in other Arab countries [12]. This is in contrast to Western world data where BC occurs at a comparatively older age, as reported to be 61 years in the United States cohort [2]. Our findings of age not being a predictor of pCR are in line with a similar study from the Middle East with a relatively younger cohort (mean age of 45.4 years) who also did not find an association of age with pCR [13]. The average age of the cohort reported by a similar European study assessing the pCR post-NAC in LABC was 55 years [14]. This indicates an earlier incidence of BC among Middle Eastern women than among Europeans. Studies have found pCR post-NAC to be associated with better prognosis, but different studies use different definitions of the pCR, interpreting results and their importance quite challenging. In this study, pCR was defined when there was an absence of invasive tumors in the breast and axilla with or without the presence of DCIS. Although it is in line with the latest definition used by most similar studies, using a standardized definition of pCR while assessing its association with the outcome is highly recommended [15]. In our study, pCR was achieved in 19.4% of the cases, which is in line with what has been reported (19.2%) in a meta-analysis of 30 studies [4]. The frequencies of ER, PR, and HER2 in our cohort were comparable to what has been previously reported [3]. Expression of ER and PR in our cohort (60.7% and 47.4%, respectively) are slightly lower than what has been reported earlier (65% and 55%, respectively). On the other hand, 33.6% of our cases had HER2 overexpression which is higher than what has been reported earlier (12-20%) [3]. We revealed statistically significant associations of negative ER, PR, and positive HER2 expression, an earlier T stage, and no LN involvement with pCR. These findings are in line with what has already been reported [16]. It has also been reported that NAC is more beneficial (higher rate of pCR) among younger BC patients because their tumors are mostly ER-negative [2]. Contrary to our findings, Miglietta L et al. and Olfatbakhsh A et al. found no association between ER and PR receptors with pCR [13, 14]. However, Miglietta L et al. reported that patients with overexpression of HER2 and T-stage 2 achieved pCR. Our finding of a statistically significant association of T stage 2 with pCR strengthens their findings. In our study, we observed a trend toward an association between pCR and higher tumor grade, which is in line with similar findings of association reported by von Minckwitz G et al. [17]. Another important immunohistochemical parameter that was found to be a statistically significant predictor of pCR in our study was the Ki67 index. Ki67 is a biomarker for the proliferation of cancer cells during the cell cycle, and NAC is known to achieve pCR in highly proliferating tumors. The mean index in our cohort was 40.9; among the pCR group, it was 48.9 compared to 31.6 in the pNR group. Our finding is strengthened by previously published similar results by Olfatbakhsh A et al., who reported an association between the Ki67 index of more than 40 with pCR [13]. Their pCR group had a mean Ki67 of 50.27 compared to the mean of 34.8 for the whole cohort. The mean Ki67 of our cohort was higher than theirs, but for the pCR group, it was almost the same. It has been reported that the threshold for high Ki67 lies at 35% [18]. Fasching PA et al. also reported a higher Ki67 index to be an independent predictor of pCR with a mean value of 50.6 [19]. 

Another important factor affecting the pCR is the molecular subtyping of invasive BC based on similarities in tumor biology and clinical outcome. The pathological response after NAC differs considerably across BC subtypes [20]. These subtypes and their biological properties have been well studied in the literature and have been accentuated even further recently with the usage of GEP [4]. However, neither is it economical to be used in common practice, nor this approach utilizes traditional pathologic features such as the number of LNs involved, margin status, primary tumor size, and others for prognostic purposes. In this study, we used the IHC-based molecular classification, which relies on IHC testing of ER, PR, HER2, and Ki67 and has been reported to have a similar prognostic and predictive value as GEP [21]. The development of guidelines regarding the testing of these prognostic markers is making the IHC-based molecular classification even more reliable. In our cohort, we reported luminal B to be the most common subtype (42.2%), followed by triple-negative (23.7%) and luminal A (19.4%), while HER2-enriched (14.7%) subtype was the least common. These findings are in line with already published data in which most cases of BC are luminal subtypes, and the least are HER2-enriched groups [13, 22]. We found molecular subtype to be significantly associated with the pathological response, with 45.2% of HER2-enriched and 28% of triple-negative subtypes developed pCR compared to only 7.3% of luminal A subtype. These findings of difference in pCR based on molecular subtypes are similar to what has been reported in a meta-analysis of 30 studies and 11695 patients where odds for achieving pCR were higher among HER2-enriched and triple-negative tumors (seven and five times, respectively) [4]. Interestingly the mean pCR rate reported in this meta-analysis was almost the same as in this study (19.2% vs 19.4%). HER2 is usually associated with more aggressive disease and a poor prognosis in 20-30% of patients with BC by making cancer cells escape from host immune surveillance [7]. In this study, the HER2-enriched subtype was the only subtype that retained its statistically significant association with pCR in logistic regression analysis, followed by triple-negative, which lost its significance. Our report that the HER2-enriched and triple-negative subtypes can be used as predictors of pCR post-NAC is in line with Rouzier R et al. [23] and further strengthened by Li XB et al., who reported a much higher mean pCR (43.9%) and a relatively higher pCR rate among HER2-enriched and triple-negative subtypes (58.2% and 47.4%, respectively) [24]. Negative HR expression in these subtypes and the fact that they are highly proliferating tumors make them more sensitive to chemotherapy compared to luminal subtypes. Further, the higher rate of pCR in the HER2-enriched subtype can be explained by the increasing use of anti-HER2 targeted therapy in the neoadjuvant settings. The triple-negative subtype accounts for approximately 20% of all BC and lacks receptor-targeted therapy due to negative HR and HER2 expression [25]. These are a heterogeneous group of tumors evident from the wide range of pathological responses observed in our study (pCR=28%, pPR=60%, and pNR=12%). They also possess a higher tumor grade, and with chemotherapy, as the only option left, achieving pCR could be used as an endpoint in establishing therapy regimens for this subtype. 

On the other hand, apart from having the lowest rate of pCR, the luminal A subtype had the highest rate of pNR (22%) in our cohort. This subtype has been found to be the least sensitive to chemotherapy [26], but still, our study population included 19.4% of patients with luminal A subtype and received NAC. The main reason for this inclusion is the fact that most of these patients were in an older age group, and other factors, like the TNM stage, played a significant role in the decision to give NAC to these patients. In line with our findings, Bonnefoi H et al. had a very similar rate of pCR (7.2%) in their patients with luminal A subtype [22], and other studies have also reported the luminal A tumors to have the least rate of pCR [4, 13]. Contrary to this, these tumors have been associated with better survival. Therefore, the Oncotype Dx recurrence score is increasingly used to predict cases with a high risk of recurrence and to identify candidates for chemotherapy. There is an increased need to validate a different set of prognostic markers to be used for patients receiving NAC to better predict the pathological response and, ultimately, the outcome. This study also assessed the impact of pathological response, molecular subtypes, and clinicopathological parameters on the clinical outcome of patients treated with NAC. We observed a 61% decrease in the risk of developing metastasis among patients who achieved pCR compared to those with pNR with statistical significance and irrespective of the molecular subtypes. Interestingly pCR was an independent predictor of better OS with 94% less risk of mortality associated with BC in patients with pCR. Spring LM et al. also reported a similar association of pCR with DFS and OS, but our findings indicate a better OS rate than theirs (93% vs. 78%; aHR: 0.07 vs. 0.22) [27]. However, the impact of molecular subtypes on DFS and OS was not demonstrated in our study. Contrary to our findings, studies have reported better outcomes in certain subtypes (HER2-enriched and triple-negative) [2, 25]. Kim H et al. found the non‐pCR group to have significantly decreased five-year OS and DFS rates compared to the pCR group, especially in triple-negative and HER2‐enriched BC patients [28]. von Minckwitz G et al. reported that in patients with luminal A subtype, pCR could not predict the outcome. Therefore pCR cannot be considered an endpoint in predicting the outcome in this specific subtype [17]. In contrast to the already reported outcomes specific to the molecular subtype, Bonnefoi H et al. reported that patients who achieve a pCR following six cycles of contemporary chemotherapy have significantly better outcomes, irrespective of their molecular subtype [22]. In contrast, outcomes were poorer when chemotherapy did not induce a pCR. This observation across all subgroups justifies the current interest in developing post-neoadjuvant trials, even in subgroups where effective targeted therapies exist.

In this study, the high Ki67 index was another factor significantly associated (p=0.006) with better DFS and showed a trend towards better OS. Contrary to our findings, in a meta-analysis of 46 studies, de Azambuja E et al. reported an inverse association of a high Ki67 index with unfavorable outcomes [18]. Our findings support the biological behavior of the tumor to be more proliferative in response to a higher Ki67 index, hence more sensitive to chemotherapy and more likely to develop pCR. On the other hand, the reason for the contrasting findings reported in the meta-analysis could be that the Ki67 index from 3.5% to 35% was considered high. In our study, patients were noticed to have less chance of developing solid organ metastasis except those who were <40 years of age, tumor stage 4 (T4), grade 3, and involvement of lymph nodes. Therefore, these factors are considered to be the reason for the unfavorable outcome (metastasis) despite achieving pCR [29]. Our findings of the younger age group associated with poor DFS are in contrast with the findings of the NSABP-18 trial, where no significant difference was found in the outcome among patients younger or older than 50 years of age [30]. Although our study has limitations due to the relatively small sample size and some selection bias due to its retrospective nature and variable NAC regimens for different patients, its strengths include a relatively younger study population with a long follow-up and the fact that it evaluated the DFS and OS along with factors influencing the pCR.

## Conclusions

In conclusion, HER2-enriched and triple-negative BC was associated with a higher rate of pCR post-NAC. Patients with pCR were less likely to develop metastasis and had significantly better OS compared to cases with pNR. Younger age, advanced stage, higher grade, and LN involvement were risk factors for metastasis.
